# Long COVID Syndrome: Lesson Learned and Future Implications

**DOI:** 10.3390/jcm12103450

**Published:** 2023-05-13

**Authors:** Giampiero Mazzaglia

**Affiliations:** Center for Public Health Research (CESP), Department of Medicine and Surgery, University of Milan-Bicocca, I-20900 Monza, Italy; giampiero.mazzaglia@unimib.it

## 1. Introduction

Severe acute respiratory syndrome coronavirus 2 (SARS-CoV-2), the virus responsible for coronavirus disease 2019 (COVID-19), has caused severe illness and mortality on a global scale, with an impact not witnessed since the 1918–19 Spanish influenza pandemic [1]. Long-term symptoms following the acute phase of COVID-19 (i.e., long COVID) were observed since the first phases of the epidemic [2] and have been documented in various studies conducted since 2020, also published in the *Journal of Clinical Medicine* [3,4].

Reportedly, around 65 million individuals worldwide have long COVID. This estimate is based on an incidence rate of 10% those infected with COVID-19 and more than 600 million recorded cases of COVID-19 [5]. Recent reviews investigating the prognostic factors associated with the risk of developing long COVID found significant association with acute COVID-19 severity, female gender, and previous comorbidities such as obesity, pulmonary diseases, and diabetes mellitus [6,7]. Conversely, the role of old age, a relevant risk factor regarding severe outcomes in COVID-19 patients, is still controversial. Two reviews [8,9] found higher risk of long COVID-19 symptomatology associated with old age, whereas the meta-analyses by Maglietta et al. [6] and Notarte et al. [7] did not find any significant association.

## 2. Lesson Learned

Mechanisms contributing to the pathophysiology of long COVID include direct pulmonary and extra-pulmonary cytotoxicity and viral-independent mechanisms such as immune dysregulation and perivascular inflammation, which contribute to the disruption of the endothelial–epithelial barrier with the invasion of white blood cells (i.e., monocytes and neutrophils) and the extravasation of an exudate at tissue level. It has been observed that several organs/systems may be affected by such mechanisms:

(1) Extravasation of the exudate into the alveolar space, as observed with other forms of acute respiratory distress syndrome (ARDS), may lead to a fibrotic state and the development of pulmonary fibrosis [3];
(2) Thrombo-inflammation provokes potential hypercoagulability. The activation of the inflammatory mechanisms in this context (i.e., endothelial injury, complement and activation, platelet–leukocyte interactions, neutrophil extracellular traps, release of pro-inflammatory cytokines, and disruption of normal coagulant) are consistent with venous thromboembolism (VTE) and arterial occlusions [10,11].
(3) Mechanisms sustaining cardiovascular sequelae in post-acute COVID-19 include direct effects, down-regulation of ACE2, and immunological damage and potential inflammation of myocardium, pericardium, and the conduction system. COVID-19 may also maintain arrhythmias due to an increased catecholaminergic state resulting from IL-6, IL-1, and tumor necrosis factor [11].

The interplay of the above-mentioned systemic derangements may also contribute to the pathologic changes seen in the central nervous system, the endocrine and dermatological manifestations, and the alteration of the gut microbiome [1].

Long COVID patients include a heterogeneous group; they range from subjects with frailty and organ damage following admissions in intensive care unit (ICU) to those with mild COVID-19 but persistent organ damage or those with a spectrum of remitting-relapsing symptoms such as fatigue, ‘’brain fog”, weakness, or chronic pain, which significantly affect the quality of life (QoL) after recovery.

The World Health Organization (WHO) published a clinical case definition of long COVID. However, still, there are neither diagnostic criteria to define patients with such a condition nor agreements in the health outcomes or symptoms that should be measured in people living with this disease. The WHO, in fact, acknowledges that “while common symptoms of long COVID can include fatigue, shortness of breath and cognitive dysfunction over 200 different symptoms have been reported that can have an impact on everyday functioning”.

To ensure that the most important outcomes/symptoms are assessed consistently, a collection of outcomes which should be measured and reported (i.e., a “core set”) in adults with post-COVID-19 conditions in clinical research and practice has been suggested. They include: (1) fatigue; (2) pain; (3) post-exertion symptoms; (4) work or occupational and study changes; (5) survival; and (6) clusters of symptoms and conditions for cardiovascular, respiratory, neurological, cognitive, mental health, and physical outcomes. Muscle and joint symptoms and conditions did not meet the a priori consensus criteria for inclusion in the core set, although they were rated of high importance among patients with long COVID [12].

The timeframe of symptoms healing is non-linear, and a complete picture of the natural history of long COVID-19 disease is still unclear. Most of the present studies address mortality and morbidity outcomes at one year after COVID-19 infection. However, a recent study [13], investigating the association between factors related to the acute phase of the disease and the presence of residual symptoms after one year from hospitalization, reported a persistence of post-COVID symptoms in up to 60% of the patient population at a mean follow-up of 17 months. The most frequent symptoms were fatigue and dyspnea, both neuropsychological disturbances.

## 3. Future Implications

Given the potential burden estimates of long COVID, the health of millions might be affected. This population of patients seeks medical advice, stresses already-overwhelmed healthcare systems, and has the potential to exacerbate the fragmentation of care.

Although the research into long COVID has been expansive and has accelerated, the actual evidence is not sufficient to provide solid guidance on treatment recommendations aimed to improve outcomes for people with long COVID. Clinical guidelines still focus on symptoms’ management, and various treatment options are being evaluated.

In addition, healthcare professionals involved in the care of acute COVID-19 survivors are likely to oversee, after hospital discharge, the recognition, documentation, investigation, and management of new symptoms, as well the follow-up of organ-specific complications developed during acute illness. Moreover, the evaluation and tracking over time of the impact of long COVID-19 on clinical, cognitive, psychological, and behavioral domains is not coordinated, thus yielding to the relevant implications for rehabilitation and long-term assistance planning [14].

There are some positive examples [15] of clinical studies showing beneficial effects of rehabilitation through therapy based on aerobic, respiratory, and low-load strength exercises, which seems to be an effective and safe strategy for the recovery of some post-COVID-19 sequelae.

However, the information is still sparce and fragmented and more randomized controlled studies are needed to ensure an adequate level of evidence across the full set of core symptoms and outcomes that characterize long COVID.

To ensure an adequate response to the long COVID crisis, we need a more comprehensive approach that contemplates the following: (1) research that builds on existing knowledge and engages patient experience in clinical studies; (2) professional societies and government agencies that develop training and education programs for the relevant healthcare stakeholders; and (3) public communication campaigns informing about long COVID and its risks.

To avoid the challenges we have witnessed in the care of the acute phase of the disease, the following requirements may be considered:

(1) A thorough estimate on the impact of long COVID in terms of the clinical and social needs of the affected individuals, their access to healthcare services, and their healthcare resource use. Several countries have set up specialized clinics [16] and patient support groups have been developed to improve the general understanding of long COVID and identify the needs of healthcare systems. However, to plan appropriate healthcare services and streamline the allocation of public health resources, it is essential to determine the burden and the needs of affected individuals;
(2) A clear definition of long COVID, a common diagnostic–therapeutic protocol, and a harmonization of outcome measures to streamline the care of these patients [17]. As previously mentioned, an attempt to harmonize case definition and clinical diagnosis has been successfully made with the creation of the core outcomes set;
(3) An interdisciplinary cooperation for comprehensive care in the outpatient setting, including rehabilitation services and telemedicine (TH). Recent experiences strongly support the vision that a coordinated approach which involves screening evaluation via TH consultation, followed by a multidisciplinary assessment in outpatient clinics, is likely the most effective way to manage the complexity of long COVID. It is also crucial for healthcare systems and hospitals to establish a coordinating framework, where specialists from multiple disciplines can provide integrated care;
(4) The involvement and integration in the COVID-19 teams (and in the patients’ care) of primary care physicians (PCP) because of their central role in the management of outpatients’ medical and non-medical needs and their ability to provide holistic support (while avoiding over-investigations) and proper access to secondary and tertiary care [18]. In fact, the effective management of long COVID requires recognition of the condition and proper consideration of the patient’s experience. It also requires a skilled, generalist assessment of the multisystem disorder and judicious referral to specialists’ services as appropriate.

Therefore, a holistic and evidenced-based approach to medical care beyond specialist care is required, and plans must be made to accommodate the needs of long COVID patients in the healthcare system.

## Data Availability

Not applicable.
